# The roles of subcellular Argonaute 2 in cardiovascular diseases

**DOI:** 10.1515/jtim-2025-0036

**Published:** 2025-07-30

**Authors:** Yifan Cai, Weijian Hang, Rong Xie, Huaping Li, Chen Chen, Feng Wang

**Affiliations:** Division of Cardiology, Department of Internal Medicine, Tongji Hospital, Tongji Medical College, Huazhong University of Science and Technology, Wuhan, Hubei Province, China; Hubei Key Laboratory of Genetics and Molecular Mechanisms of Cardiological Disorders, Tongji Hospital, Tongji Medical College, Huazhong University of Science and Technology, Huazhong University of Science and Technology, Wuhan, Hubei Province, China

**Keywords:** Argonaute 2, subcellular localisation, gene regulation, cardiovascular diseases

## Abstract

Argonaute 2 (Ago2), the core component of the microRNA-induced silencing complex (miRNA-RISC), is a pivotal protein with a well-established and potent role in gene expression regulation. Traditionally, Ago2 functions at the post-transcriptional level by binding to non-coding RNAs in the cytoplasm, facilitating gene expression via cleavage, deadenylation, or repression of target messenger RNA (mRNA) translation. Emerging evidence indicates that Ago2 can be transported from the cytoplasm to the nucleus or mitochondria, where it performs its critical functions. We observed that nuclear and mitochondrial Ago2 have been increasingly implicated in the pathogenesis of various cardiovascular diseases, such as hypertension, diabetic cardiomyopathy, and heart failure. These findings suggest a potential novel therapeutic strategy for targeting Ago2 in cardiovascular conditions. In this review, we aim to provide a comprehensive overview of recent studies elucidating the transport mechanisms of mammalian Ago2 into various subcellular organelles and summarise the functional roles and molecular mechanisms of subcellular Ago2 in cardiovascular diseases, offering a theoretical framework for Ago2-related therapeutic strategies.

## Introduction

Argonaute 2 (Ago2), first identified in *Drosophila*, is a protein that plays a pivotal role in regulating gene expression.^[1]^ Ago2 primarily functions by binding to small non-coding RNAs (sncRNAs) —including microRNAs (miRNA), small interfering RNAs (siRNA), PIWI-interacting RNAs (piRNA), and transfer RNA-derived small RNAs (tsRNA)^[2,3]^—to interact with target DNA or nascent RNA adjacent to the target DNA. While most research has traditionally focused on the cytoplasmic function of Ago2, its broader biological roles are now emerging. Ago2 is associated with double-stranded siRNA or miRNA processed by Dicer, retaining the guide strand^[4]^ to form the RNA-induced silencing complex (RISC) through the interaction with GW182.^[5]^ This complex binds specifically to the 3’ untranslated regions of target mRNA, mediating gene silencing or downregulation *via* mechanisms such as mRNA cleavage, deadenylation, and translational repression. This process is known as post-transcriptional gene silencing (PTGS) or RNA interference (RNAi).^[1]^

Recently, increasing evidence has demonstrated the critical functions of Ago2 in the nucleus and mitochondria. Studies have shown that Ago2 can be transported from the cytoplasm to the nucleus or mitochondria, accompanied by shifts in the distribution of sncRNAs and associated protein complexes. Within these subcellular compartments, Ago2 performs several complex functions, including the activation or inhibition of target gene expression at the transcriptional or post-transcriptional levels. These mechanisms are implicated in the molecular pathology of various cardiovascular conditions, such as diabetic cardiomyopathy, atherosclerosis, hypertensive cardiomyopathy, and heart failure.^[6, 7, 8, 9, 10]^ This review aims to comprehensively analyze the latest discoveries to summarise the transport mechanisms by which the subcellular Ago2 performs within mammalian cells and integrate the findings on its role in cardiovascular diseases (CVDs).

## Nuclear localization and functional mechanisms of ago2

Nuclear Ago2, identified across various species, serves multiple functions, including RNAi, transcriptional repression or activation,^[11,12]^ chromatin topology regulation,^[13,14]^ DNA repair^[15,16]^ and alternative splicing.^[17,18]^ This review focuses on research involving mammals to provide a representative understanding of the mechanisms underlying nuclear Ago2.

### Distribution and translocation of Ago2 in the nucleus

Since the initial report of mammalian nuclear Ago2 in 2006,^[11]^ subsequent research has confirmed its localization in the nuclei of various mammalian cells.^[19, 20, 21]^ In a recent study involving 12 human cancer cell lines, nuclear Ago2 levels were found to range from 0% to 60%,^[22]^ indicating intricate regulatory mechanisms. The nuclear localization of Ago2 can vary under physiological and pathological conditions. such as diabetes, heart failure,^[6,9]^ cell density, proliferation, ageing, and stress.^[23, 24, 25, 26]^

These findings suggest the existence of a dedicated nuclear transport mechanism for Ago2. Evidence suggests that Ago2 shuttles between the nucleus and cytoplasm by binding to sncRNAs.^[20]^ Several proteins have been implicated in this transport process, including Imp8, F-actin, GW182, Mex3a, FAM172A, and Lamin A.^[10,19,22,27, 28, 29, 30]^ GW182, a key cytoplasmic component of PTGS, interacts with Ago2 and may allow for reciprocal modulation of its nuclear localization.^[28,29]^ Mex3a forms a ternary complex by binding with Ago2 and miRNAs on the outer membrane of autophagosomes, facilitating the translocation of Ago2 into the nucleus.^[10]^ Additionally, the absence of Lamin A has been demonstrated to trigger the nuclear translocation of Ago2, which could be attributed to the reduction in nuclear lamina rigidity.^[22]^ Despite these advances, a universally accepted transport mechanism for nuclear Ago2 remains elusive.

### The role of nuclear Ago2 in transcriptional control

Mammalian nuclear Ago2 has been identified as a vital component in transcriptional gene silencing (TGS) and gene activation modulation.^[11,31]^ Previous research has provided compelling evidence that Ago2 depletion can reverse sncRNA-induced gene silencing or activation.^[32]^ For example, our research showed that nuclear Ago2 can effectively restore miR-320-mediated transcriptional enhancement of CD36 in Ago2-depleted cardiomyocytes, whereas cytoplasmic Ago2 fails to rescue this effect.^[9]^ Collectively, these observations underscore the indispensable role of nuclear Ago2 in regulating gene expression.

#### Promoter activity regulation

Recent studies have begun to unravel the underlying regulatory mechanisms of Ago2 at promoters. In the cytoplasm, Ago2 is integrated with sncRNAs to construct an RISC, selectively targeting designated molecules. However, other integral components, such as DICER and TRBP, are not imported into the nuclear compartment with Ago2.^[20]^ The targeting mechanism of nuclear Ago2 remains a topic of debate. Some studies suggest that nuclear Ago2 directly targets promoter regions,^[33, 34, 35]^ whereas others propose that it targets transcripts near promoter regions, such as pRNA.^[32,36, 37, 38]^ Our prior work provided a comprehensive explanation for this discordant phenomenon, demonstrating that pRNA facilitates sncRNA binding to DNA and enables Ago2 to associate with sncRNAs and the corresponding promoter DNA. The pRNA itself may harbor binding sites, allowing sncRNAs to associate with it. Without these pRNA binding sites, sncRNAs compete with pRNA for the same binding loci on the DNA.^[39]^

Once bound, Ago2 and sncRNA can either activate or repress transcription near promoters, as observed at multiple gene loci in cancer cells and mammalian cardiomyocytes. The genetic regulatory effect of Ago2 and sncRNAs appears to be influenced by an array of intricate factors, including the differential levels of target gene expression, sequence discrepancies,^[12]^ and the orientation of sense and antisense promoter RNAs.^[39]^ However, the role of Ago2 is fundamentally analogous to transcriptional repression or activation processes.

#### Methylation modification at promoters

Recently, a transcriptional regulatory process involving Ago2 and the sncRNA complex utilizing DNA or pRNA as a platform to recruit chromatin-modifying proteins, facilitating the activation or repression methylation marks of histones,^[40,41]^ has been widely investigated. Previous studies have suggested that nuclear Ago2 participates in the formation of repression markers, such as H3K9me2,^[23,41]^ H3K9me3,^[31,40]^ and H3K27me3,^[23,33,40,42]^ as well as activation markers, such as H3K4me3 and H2Bub1 (histone 2B ubiquitination),^[23,35]^ which can stimulate the methylation of H3K4 in humans.^[43]^ Repressive methylation signatures, such as H3K9me3, recruit heterochromatin protein 1 (HP1),^[44]^ promoting chromatin conformational changes that facilitate heterochromatin assembly and attenuate transcriptional activity.^[45]^ Conversely, certain methylation markers, such as H3K4me3, enhance gene transcription by counteracting the heterochromatic modifications, as well as modulating transcriptional pause/release and elongation processes.^[46]^ Although DNA methylation is a common epigenetic mechanism for gene silencing, it has yet to be directly attributed to Ago2 in mammalian cells.^[40,41]^

#### Noncanonical regulation of Ago2 in the nucleus

The aforementioned mechanisms are the primary focus of current studies. Beyond these established mechanisms, Ago2 has been implicated in alternative regulatory pathways. For instance, Ago2 positioned at promoter sites can inhibit transcription initiation by reducing RNA Pol II and TFIIB localization at the promoter, independent of known epigenetic modifications.^[37]^ Furthermore, Ago2-sncRNAs complexes can target not only promoters but also terminators,^[47]^ 3’ ends of genes,^[42]^ short tandem intergenic region (STIR),^[48]^ in addition to enhancers,^[49]^ where they perform diverse roles, including histone acetylation. Moreover, novel sncRNAs, such as switch/sucrose non-fermentable complex-interacting RNA (swiRNA)^[14]^ and tsRNA,^[3]^ further expand Ago2’s functional repertoire. These emerging insights into the noncanonical functions of nuclear Ago2 lay the groundwork for future research.

## Mitochondrial localization and functional mechanisms of ago2

### Mitochondrial localization of Ago2

In addition to its well-established localization within the cytoplasm and nucleus, Ago2 has been identified in the mitochondria, as supported by several studies.^[50,51]^ The mitochondrial presence of Ago2 is finely regulated by physiological conditions and pathological stimuli, such as cell differentiation^[52]^ and diabetic damage.^[7,53]^ Our findings indicate that both type I and type II diabetes can significantly reduce mitochondrial Ago2 levels in cardiomyocytes. Similarly, cardiomyocytes from diabetic patients with heart failure exhibit decreased mitochondrial Ago2 content.^[7]^ Our recent research elucidated the transport mechanisms governing mitochondrial Ago2 in the context of diabetes. Diabetes-induced reductions in SIRT3 levels in cardiomyocytes lead to increased malonylation of cytoplasmic Ago2, impairing its interaction with Timm17b. This disruption ultimately inhibits the translocation of Ago2 into the mitochondria.^[7]^

### Mitochondrial translation regulation by Ago2

Current research suggests that mitochondrial Ago2 serves multiple roles, including the regulation of translation and transcription,^[52,54,55]^ sncRNA biogenesis,^[56]^ and sncRNA transport.^[57]^ Mitochondrial Ago2-mediated translation mirrors cytoplasmic RNAi, a process that requires its association with sncRNAs, primarily MitomiRs.^[50]^ Previous studies have shown that mitochondrial Ago2 is involved in the translational repression of cardiomyocytes and RNAi in cancer cells,^[54,58]^ with the latter mechanism dependent on Ago2’s endonucleolytic splicing activity. Beyond translational inhibition, discoveries by Zhang *et al*. suggest that the Ago2-miR-1 complex promotes mitochondrial translation of ND1 and COX1 by binding with specific mitochondrial mRNAs in mouse myoblasts. In addition, our earlier findings revealed that miR-21 enhances Cytb translation by strengthening the interaction between mitochondrial Ago2 and Cytb mRNA, underscoring the instrumental role of Ago2 in mitochondrial translational.^[8]^ Furthermore, we discovered that Ago2 recruits the mitochondrial Tu translation elongation factor (TUFM), facilitating translational elongation and providing new insights into its regulatory functions.^[7]^

Despite parallels between Ago2-mediated translational regulation in the mitochondria and cytoplasm, the underlying mechanisms may differ. For example, the mitochondrial RISC exhibits distinct features in terms of composition. At least three distinctive studies have established that GW182 is essential for Ago2-mediated translational control in the mitochondria.^[8,52,53]^ Another RISC component is DICER, which can be detected in the mitochondria of rat hippocampal and human osteosarcoma cells ^[56,59]^ but not in rat cardiomyocytes.^[53,54]^ Conversely, the RISC component FXR1, which can be found in the mitochondria of rat cardiomyocytes, is highly correlated with translational inhibition of mitochondrial Ago2^[53]^ but has an opposing effect in the cytoplasm.^[60]^ These findings highlight the unique regulatory framework of Ago2 in mitochondrial translation, which may be distinct from that of the cytoplasm.

### Alternative functional roles of mitochondrial Ago2

In addition to translational regulation, mitochondrial Ago2 contributes to transcriptional inhibition, tsRNA synthesis, and miRNA shuttling. Song *et al*. demonstrated that miR-2392 is associated with Ago2 and mitochondrial DNA (mtDNA) genes, antagonizing transcription.^[55]^ In our previous study, we identified that Ago2 overexpression leads to elevated levels of specific miRNAs and their precursor miRNAs.^[7]^ These findings suggested the potential role of Ago2 in mitochondrial transcription. Ago2 may also be involved in the biogenesis of other non-coding RNAs within the mitochondria. For instance, Meseguer *et al*. demonstrated that mitochondrial Ago2 collaborates with Dicer to generate mt-tRNA-derived fragments and mt-tsRNAs in mitochondrial encephalomyopathy, lactic acidosis, and strokelike episodes (MELAS) syndrome.^[56,61]^ Additionally, Ago2 has been proposed as a potential mediator of miRNA translocation, as it is associated with a pathway through polynucleotide phosphorylase (PNPase), facilitating the translocation of miRNA-378 into the mitochondria.^[57]^

## Subcellular mechanistic effects of ago2 in CVDS

**Figure 1:**
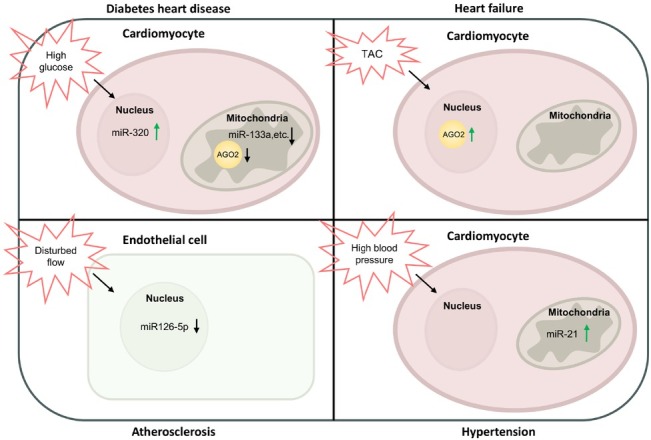
Expressional changes of subcellular Ago2/miRNAs in CVDs. Under diabetes, high glucose upregulates nuclear miR-320 expression in cardiomyocytes. In the mitochondria of cardiomyocytes, high glucose decreases Ago2 and miR-133a expression. Under TAC induced heart failure, nuclear Ago2 is increased in cardiomyocytes. In atherosclerosis, disturbed blood flow reduces miR-126-5p expression in the nucleus of endothelial cells. Under hypertension, high blood pressure increases miR-21 expression in the mitochondria of cardiomyocytes. TAC: transverse aortic constriction; Ago2: Argonaute 2; miRNA: microRNA; CVDs: cardiovascular diseases.

The sub-cellular role of Ago2 in CVDs is multifaceted, encompassing processes such as cell apoptosis, cardiomyocyte differentiation, lipid deposition, reactive oxygen species (ROS) production, and abnormal adenosine triphosphate (ATP) synthesis. Collectively, these mechanisms contribute to the development of atherosclerotic plaques, cardiac remodeling, impaired myocardial contractility, and metabolic dysregulation. The expressional changes of subcellular Ago2 and/or miRNAs in various CVDs are shown in Figure 1.

### Diabetic heart disease

The involvement of subcellular Ago2 in diabetic cardiomyopathy is dual-faceted and can be summarised as follows: First, Ago2 suppresses ATP synthesis by inhibiting the production of mitochondrial ATP synthase subunits. Second, nuclear Ago2 enhances the fatty acid uptake in the heart, leading to cardiac lipotoxicity and myocardial metabolic imbalances. Finally, mitochondrial Ago2 plays a cardioprotective role by stabilizing the mitochondrial electron transport chain and reducing ROS production. In diabetic cardiomyocytes, mitochondrial Ago2 levels are significantly reduced.^[53,62]^ Nevertheless, in type 1 diabetes, the increased miR-378 that inhibits ATP6 synthesis, in conjunction with Ago2, mediates the translational blockade of ATP6, diminishing the catalytic efficacy of ATP synthase. This may affect the energy supply for cardiomyocytes, where mitochondrial oxidative phosphorylation accounts for approximately 95% of the ATP production required for cardiomyocytes.^[63]^ In addition, mitochondrial Ago2 may also play a role in myocardial protection. The results of our previous study demonstrated that under diabetic stress, the miRNAs targeting mt-Cytb and mitochondrial Ago2 expression are reduced, disrupting electron transport chain complex III (ETCIII) subunit assembly, and leading to excessive ROS production.^[7]^ This exacerbates myocardial stiffness and injury through oxidative stress, mitochondrial dysfunction, inflammatory cascades, and fibroblast activation.^[64,65]^ Nuclear Ago2 also plays a crucial role in diabetic cardiomyopathy. Our research verified that under diabetic injury, the elevated miR-320, together with Ago2 and RNA Polymerase II, promotes CD36 transcription, while Ago2 stabilizes miR-320 throughout this process.^[9]^ CD36 is a free fatty acid transporter on the surface of cardiomyocytes.^[66]^ Elevated CD36 expression accelerates cardiomyocyte lipid deposition, contributing to the progression of diabetic cardiomyopathy by generating excessive ROS, altering metabolic balance, and promoting apoptosis.^[67,68]^

### Atherosclerosis

The pathogenesis of atherosclerosis is closely associated with hemodynamic alterations. While sustained laminar flow and high shear stress in the linear segments of the arteries enhance the expression of endothelial-protective proteins, turbulent flow and low shear stress in arterial curves and bifurcations exert an antagonistic influence.^[69]^ Previous studies have shown that Ago2 can be involved in the hemodynamic abnormalities underlying atherosclerosis.^[10]^ Caspase-3 is an effector molecule involved in the nuclear changes during apoptosis, including DNA fragmentation, chromatin condensation, and nuclear disruption.^[70]^ The application of high shear stress has been demonstrated to increase autophagy and localization of miR-126 in the nucleus, where miR-126 inhibits apoptosis by directly reducing the cleavage of caspase-3. During this process, Ago2 forms a ternary complex with Mex3a and miR-126 on the surface of autophagosomes and translocates to the nucleus. Subsequently, Ago2 dissociates from the miR-126 in the nucleus, allowing miR-126 to bind to caspase-3.^[10]^ Dysfunctional efferocytosis and the accumulation of apoptotic endothelial and foam cells collectively accelerate atherosclerotic plaque formation.^[71]^ These findings indicate that Ago2 may play a protective role against atherosclerosis.

### Hypertension

Our previous study has shown that during hypertensive stress, the compensatory elevation of mitochondrial miR-21 in cardiomyocytes promotes the biosynthesis of mt-Cytb by increasing the association of Ago2 with mt-Cytb mRNA, thereby ensuring the structural integrity of ETCIII and mitigating the generation of ROS inside the mitochondria. It is well established that ROS contributes to hypertension by impairing vasodilation, increasing vascular resistance and cellular proliferation, activating matrix metalloproteinases (MMPs), and promoting extracellular matrix protein deposition, *etc*.^[72,73]^ Notably, the knockdown of GW182 did not affect this translation process, suggesting that the translational regulatory mechanism of Ago2 in mitochondria may be different from that in the cytoplasm.^[8]^

### Heart failure

Recently, we confirmed that Ago2 is intricately involved in the pathological processes of heart failure, particularly through its role in cardiac remodeling and functional impairment. In a mouse model of heart failure induced by transverse aortic constriction, an increase in Ago2 levels was observed in both the cytoplasm and nuclei of cardiomyocytes, resulting in a detrimental impact on cardiac function. This effect is multi-stage, exerting influence during both the compensated and decompensated phases of heart failure. Further investigation revealed that the underlying mechanism is the transcriptional activation of ankyrin repeat domain-containing protein 1 (ANKRD1) mediated by Ago2 and miRNA interactions, which exerts cardioprotective effects in the cytoplasm and causes pathological remodeling by altering the myosin heavy chain isotype 7 to 6 ratio (*MYH7/MYH6* ratio) in the nucleus.^[74, 75, 76]^ Despite Ago2 increasing ANKRD1 levels in both subcellular compartments, the overall impact of increased ANKRD1 is generally detrimental to cardiac function.

### Subcellular Ago2 in non-cardiovascular systems

Some pathways of Ago2, although not directly demonstrated to be involved in the process of CVD, may have potential effects on CVD. For example, our unpublished preliminary data revealed that liver mitochondrial Ago2 overexpression attenuated glucose and lipid disorders. Besides, early studies on the subcellular mechanisms of Ago2 focused on the effects on progesterone receptor (PR),^[11,32,42]^ which may be involved in regulating the process of cardiomyocyte development and maturation.^[77]^ In addition, most non-cardiovascular studies have focused on the effects of Ago2 on cancer, some of which include non-classical pathways of Ago2, such as prostate cancer,^[37]^ and osteosarcoma.^[49]^ Cancer and CVD share many common mechanisms and risk factors,^[78]^ and many cancer therapies have been shown to have cardiovascular toxicity.^[79,80]^ The systemic investigation of subcellular Ago2/miRNAs machinery in non-cardiovascular tissues are intriguing subject for further research.

## Therapeutic strategies and challenges

Studies of the subcellular mechanisms of ago2 have revealed many therapeutic approaches targeting the Ago2/miRNA complex as well as its downstream molecules. Considering the Food and Drug Administration (FDA) approval of recombinant adeno-associated virus (rAAV) drugs Luxturna and Zolgensma, exogenous miRNAs or Ago2 can be delivered using rAAV9 as a vector. For example, tail vein injection of rAAV-miR-21 can alleviate pathological injury in spontaneous hypertension rats.^[8]^ Exogenous delivery of mitochondrial-targeted rAAV9-Ago2 can prevent cardiac dysfunction in diabetic mice.

In our unpublished research (Patent Number: ZL20221 1079886.4), rAAV9-mitochondrial Ago2 also protected against transverse aortic constriction (TAC) pressure overload induced heart failure. Owing to the refinement of Ago2 subcellular localization signals and its involvement in a broad spectrum of signaling pathways, it emerges as a more advantageous therapeutic target compared to individual miRNAs. Besides, rAAV9-Ago2 short-hairpin RNA (shRNA) delivery is an applicable method to treat heart failure. Alternatively, siRNAs, are also safe for clinical application, as exemplified by the use of lipid lowering drug, Inclisiran. Targeting downstream molecules of Ago2 also represents a promising strategy. For instance, ANKRD1, which confers cardioprotection in the cytoplasm, can induce a dual effect by inhibiting its nuclear transport. However, the aforementioned strategies may entail potential side effects, such as uncontrolled immune responses presented by rAAV and siRNAs. Therefore, patients with cardiac dysfunction who are co-diagnosed with autoimmune diseases should be treated with caution.

## Conclusions and perspectives

Although nuclear Ago2 was identified early in mammals, its significance remains underappreciated. While previous investigations have highlighted several factors that may influence nuclear Ago2 levels^[6,9,23, 24, 25, 26]^ and the expression of relevant proteins,^[10,19,22,27, 28, 29, 30]^ these findings fall short of establishing a comprehensive mechanistic model. The transcriptional regulatory roles of nuclear Ago2 involve intricate mechanisms, including promoter regulations, methylation modifications, and non-classical pathways, adding layers of complexity. Similarly, mitochondrial Ago2 concentration is influenced by a spectrum of physiological and pathological factors.^[7,52,53]^ Chiefly, we identified a critical mitochondrial transport pathway for Ago2 mediated by Timm17b.^[7]^ Preliminary studies have suggested that the primary role of mitochondrial Ago2 lies in translational regulation, a function distinct from its cytoplasmic counterpart. Additionally, mitochondrial Ago2 exhibits alternative roles such as transcriptional inhibition, tsRNA generation, and miRNA shuttling.

Though the subcellular Ago2/miRNA complex was investigated in a variety of CVDs including diabetic cardiac dysfunction, hypertension and pressure overload induced heart failure, its functions in other CVDs such as myocardial infarction, and obesity cardiomyopathy are still unclear. Moreover, the same gene/protein may play different roles in different stages of diseases. In terms of Ago2, our previous study showed that in TAC induced heart failure, rAAV9-mediated nuclear Ago2 administration before or after TAC surgery both promoted heart failure,^[6]^ indicating its regulatory role in both the compensation and decompensation phase. However, for other CVDs, the role of Ago2 and/or miRNAs in different disease stages is largely unknown and awaits further investigation.

**Figure 2:**
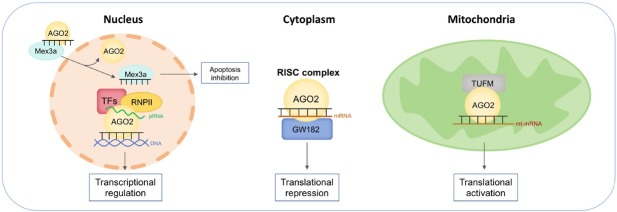
Mechanisms of Ago2/miRNAs complex in various subcellular compartments. In the nucleus, 1) miRNA dissociates from Ago2 and binds to caspase-3 in an aptamer-like fashion, preventing dimerization of the caspase and inhibiting its activity to limit apoptosis. 2) nuclear Ago2/miRNAs recruit transcriptional factors and target gene promoters to regulate gene transcription. In the cytoplasm, Ago2/miRNAs recruit GW182 to mRNA to mediate translational silencing. In the mitochondria, Ago2/miRNAs directly interact with translation elongation factor TUFM to enhance mitochondrial translation. Ago2: Argonaute 2; TUFM: the mitochondrial Tu translation elongation factor; miRNA: microRNA; RISC: RNA-induced silencing complex.

**Figure 3:**
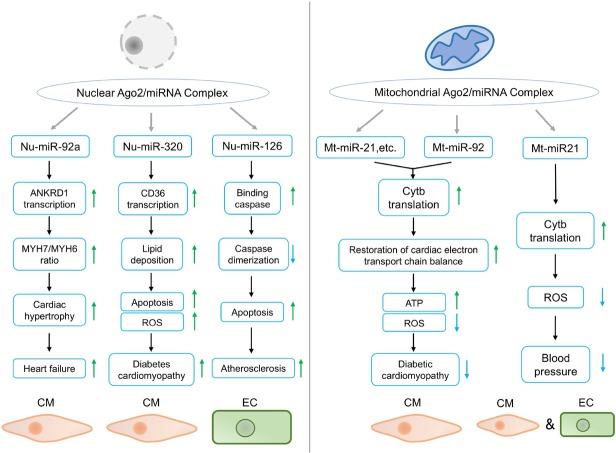
Pathophysiologic functions of nuclear and mitochondrial Ago2/miRNAs complex in CVDs. In the nucleus, miR-92a promotes ANKRD1 transcription, and increases *MYH7/MYH6* ratio in CMs, leading to cardiac hypertrophy and heart failure under TAC stress. 2) miR-320 enhances CD36 transcription, increases lipid uptake and ROS production in CM, leading to deteriorated diabetes cardiomyopathy. 3) miR-126 inhibits caspase dimerization and promotes apoptosis in ECs, contributing to atherosclerosis. In the mitochondria, 1) miR-21 and miR-92 activate Cytb translation and restore electron transport chain balance in CM, protecting against diabetic cardiomyopathy. 2) miR-21 upregulates Cytb translation, decreases ROS generation in CM and EC, lowering blood pressure. Green and blue arrows indicate activation and inhibition, respectively. CM: cardiomyocyte; TAC: transverse aortic constriction; EC: endothelial cell; Cytb: cytochrome b; ROS: reactive oxygen species; ANKRD1: ankyrin repeat domain-containing protein 1; ATP: adenosine triphosphate; CVDs: cardiovascular diseases; miRNA: microRNA.

In summary, we have drawn a diagram to illustrate the detailed mechanism of Ago2’s contrasting roles in different cellular compartments (Figure 2). Moreover, we streamlined the noncanonical functions of Ago2 in CVD development and progression (Figure 3). The noncanonical functions of nuclear and mitochondrial Ago2/miRNAs in CVDs provided new treatment strategies. Mechanistically, nuclear Ago2/miRNAs activated gene transcription while mitochondrial miRNAs enhanced mitochondrial gene translation. However, the mechanism of nuclear and mitochondrial Ago2/miRNAs is not fully understood: (1) Previous work demonstrated that nuclear Ago2 recruited a unique set of proteins, including CTR9, CTR9 is a component of PAF1C, which is required for the phosphorylation of Ser2 of RNA polymerase II C-terminal domain, to form a small activating RNA-induced transcriptional activation complex in cancer PC-3 cell line,^[35]^ whether this is also the case for nuclear Ago2 in cardiomyocytes during the development and progression of CVDs is unclear. (2) As for mitochondrial Ago2, we have revealed that Ago2/miRNAs directly interacted with TUFM to enhance mitochondrial translation. However, the direct binding sites between Ago2 and TUFM are unclear. (3) In terms of the role of subcellular Ago2, it seemed that short-term treatment (2-6 months) by rAAV improved cardiac performance, however, whether longer-term treatment or higher dosage treatment would lead to similar effects is unclear. (4) the driving causes of subcellular Ago2 remodeling during various CVDs are largely unknown.

## References

[1] Hammond SM, Boettcher S, Caudy AA, Kobayashi R, Hannon GJ (2001). Argonaute 2, a link between genetic and biochemical analyses of RNAi. Science.

[2] Carthew RW, Sontheimer EJ (2009). Origins and mechanisms of miRNAs and siRNAs. Cell.

[3] Di Fazio A, Schlackow M, Pong SK, Alagia A, Gullerova M (2022). Dicer dependent tRNA derived small RNAs promote nascent RNA silencing. Nucleic Acids Res.

[4] Meister G, Tuschl T (2004). Mechanisms of gene silencing by double-stranded RNA. Nature.

[5] Jakymiw A, Lian S, Eystathioy T, Li S, Satoh M, Hamel JC (2005). Disruption of GW bodies impairs mammalian RNA interference. Nat Cell Biol.

[6] Xie R, Yuan S, Hu G, Zhan J, Jin K, Tang Y (2024). Nuclear AGO2 promotes myocardial remodeling by activating ANKRD1 transcription in failing hearts. Mol Ther.

[7] Zhan J, Jin K, Xie R, Fan J, Tang Y, Chen C (2024). AGO2 protects against diabetic cardiomyopathy by activating mitochondrial gene translation. Circulation.

[8] Li H, Zhang X, Wang F, Zhou L, Yin Z, Fan J (2016). microRNA-21 lowers blood pressure in spontaneous hypertensive rats by upregulating mitochondrial translation. Circulation.

[9] Li H, Fan J, Zhao Y, Zhang X, Dai B, Zhan J (2019). Nuclear miR-320 mediates diabetes-induced cardiac dysfunction by activating transcription of fatty acid metabolic genes to cause lipotoxicity in the heart. Circ Res.

[10] Santovito D, Egea V, Bidzhekov K, Natarelli L, Mourão A, Blanchet X (2020). Noncanonical inhibition of caspase-3 by a nuclear microRNA confers endothelial protection by autophagy in atherosclerosis. Sci Transl Med.

[11] Janowski BA, Huffman KE, Schwartz JC, Ram R, Nordsell R, Shames DS (2006). Involvement of AGO1 and AGO2 in mammalian transcriptional silencing. Nat Struct Mol Biol.

[12] Janowski BA, Younger ST, Hardy DB, Ram R, Huffman KE, Corey DR (2007). Activating gene expression in mammalian cells with promoter-targeted duplex RNAs. Nat Chem Biol.

[13] Nazer E, Dale RK, Chinen M, Radmanesh B, Lei EP (2018). Argonaute2 and LaminB modulate gene expression by controlling chromatin topology. PLoS Genet.

[14] Carissimi C, Laudadio I, Cipolletta E, Gioiosa S, Mihailovich M, Bonaldi T (2015). ARGONAUTE2 cooperates with SWI/SNF complex to determine nucleosome occupancy at human Transcription Start Sites. Nucleic Acids Res.

[15] Jiang J, Ou X, Han D, He Z, Liu S, Mao N (2022). A diRNA-protein scaffold module mediates SMC5/6 recruitment in plant DNA repair. Plant Cell.

[16] Wei W, Ba Z, Gao M, Wu Y, Ma Y, Amiard S (2012). A role for small RNAs in DNA double-strand break repair. Cell.

[17] Taliaferro JM, Aspden JL, Bradley T, Marwha D, Blanchette M, Rio DC (2013). Two new and distinct roles for Drosophila Argonaute-2 in the nucleus: alternative pre-mRNA splicing and transcriptional repression. Genes Dev.

[18] Liu J, Hu J, Corey DR (2012). Expanding the action of duplex RNAs into the nucleus: redirecting alternative splicing. Nucleic Acids Res.

[19] Weinmann L, Höck J, Ivacevic T, Ohrt T, Mütze J, Schwille P (2009). Importin 8 is a gene silencing factor that targets argonaute proteins to distinct mRNAs. Cell.

[20] Ohrt T, Mütze J, Staroske W, Weinmann L, Höck J, Crell K (2008). Fluorescence correlation spectroscopy and fluorescence cross-correlation spectroscopy reveal the cytoplasmic origination of loaded nuclear RISC in vivo in human cells. Nucleic Acids Res.

[21] Tan GS, Garchow BG, Liu X, Yeung J, Morris JP, Cuellar TL (2009). Expanded RNA-binding activities of mammalian Argonaute 2. Nucleic Acids Res.

[22] Lobo V, Nowak I, Fernandez C, Correa Muler AI, Westholm JO, Huang HC (2024). Loss of Lamin A leads to the nuclear translocation of AGO2 and compromised RNA interference. Nucleic Acids Res.

[23] Benhamed M, Herbig U, Ye T, Dejean A, Bischof O (2012). Senescence is an endogenous trigger for microRNA-directed transcriptional gene silencing in human cells. Nat Cell Biol.

[24] Castanotto D, Zhang X, Alluin J, Zhang X, Rüger J, Armstrong B (2018). A stress-induced response complex (SIRC) shuttles miRNAs, siRNAs, and oligonucleotides to the nucleus. Proc Natl Acad Sci USA.

[25] Sala L, Kumar M, Prajapat M, Chandrasekhar S, Cosby RL, La Rocca G (2023). AGO2 silences mobile transposons in the nucleus of quiescent cells. Nat Struct Mol Biol.

[26] Johnson KC, Kilikevicius A, Hofman C, Hu J, Liu Y, Aguilar S (2024). Nuclear localization of Argonaute 2 is affected by cell density and may relieve repression by microRNAs. Nucl Acids Res.

[27] Ahlenstiel CL, Lim HG, Cooper DA, Ishida T, Kelleher AD, Suzuki K (2012). Direct evidence of nuclear Argonaute distribution during transcriptional silencing links the actin cytoskeleton to nuclear RNAi machinery in human cells. Nucleic Acids Res.

[28] Schraivogel D, Schindler SG, Danner J, Kremmer E, Pfaff J, Hannus S (2015). Importin-beta facilitates nuclear import of human GW proteins and balances cytoplasmic gene silencing protein levels. Nucleic Acids Res.

[29] Nishi K, Takahashi T, Suzawa M, Miyakawa T, Nagasawa T, Ming Y (2015). Control of the localization and function of a miRNA silencing component TNRC6A by Argonaute protein. Nucleic Acids Res.

[30] Sallis S, Bérubé-Simard FA, Grondin B, Leduc E, Azouz F, Bélanger C (2023). The CHARGE syndrome-associated protein FAM172A controls AGO2 nuclear import. Life Sci Alliance.

[31] Li LC, Okino ST, Zhao H, Pookot D, Place RF, Urakami S (2006). Small dsRNAs induce transcriptional activation in human cells. Proc Natl Acad Sci USA.

[32] Chu Y, Yue X, Younger ST, Janowski BA, Corey DR (2010). Involvement of argo-naute proteins in gene silencing and activation by RNAs complementary to a non-coding transcript at the progesterone receptor promoter. Nucleic Acids Res.

[33] Hu J, Chen Z, Xia D, Wu J, Xu H, Ye ZQ (2012). Promoter-associated small double-stranded RNA interacts with heterogeneous nuclear ribonu-cleoprotein A2/B1 to induce transcriptional activation. Biochem J.

[34] Meng X, Jiang Q, Chang N, Wang X, Liu C, Xiong J (2016). Small activating RNA binds to the genomic target site in a seed-region-dependent manner. Nucleic Acids Res.

[35] Portnoy V, Lin SH, Li KH, Burlingame A, Hu ZH, Li H (2016). saRNA-guided Ago2 targets the RITA complex to promoters to stimulate transcription. Cell Res.

[36] Schwartz JC, Younger ST, Nguyen NB, Hardy DB, Monia BP, Corey DR (2008). Antisense transcripts are targets for activating small RNAs. Nat Struct Mol Biol.

[37] Napoli S, Pastori C, Magistri M, Carbone GM, Catapano CV (2009). Promoter-specific transcriptional interference and c-myc gene silencing by siRNAs in human cells. EMBO J.

[38] Matsui M, Chu Y, Zhang H, Gagnon KT, Shaikh S, Kuchimanchi S (2013). Promoter RNA links transcriptional regulation of inflammatory pathway genes. Nucleic Acids Res.

[39] Li H, Zhan J, Zhao Y, Fan J, Yuan S, Yin Z (2020). Identification of ncRNA-mediated functions of nucleus-localized miR-320 in cardiomyocytes. Mol Ther Nucleic Acids.

[40] Cho S, Park JS, Kang YK (2014). AGO2 and SETDB1 cooperate in promoter-targeted transcriptional silencing of the androgen receptor gene. Nucleic Acids Res.

[41] Younger ST, Corey DR (2011). Transcriptional gene silencing in mammalian cells by miRNA mimics that target gene promoters. Nucleic Acids Res.

[42] Yue X, Schwartz JC, Chu Y, Younger ST, Gagnon KT, Elbashir S (2010). Transcriptional regulation by small RNAs at sequences downstream from 3’ gene termini. Nat Chem Biol.

[43] Sun ZW, Allis CD (2002). Ubiquitination of histone H2B regulates H3 methyla-tion and gene silencing in yeast. Nature.

[44] Lachner M, O’Carroll D, Rea S, Mechtler K, Jenuwein T (2001). Methylation of histone H3 lysine 9 creates a binding site for HP1 proteins. Nature.

[45] Almeida R, Allshire RC (2005). RNA silencing and genome regulation. Trends Cell Biol.

[46] Wang H, Fan Z, Shliaha PV, Miele M, Hendrickson RC, Jiang X (2023). H3K4me3 regulates RNA polymerase II promoter-proximal pause-release. Nature.

[47] Skourti-Stathaki K, Kamieniarz-Gdula K, Proudfoot NJ (2014). Proudfoot. R-loops induce repressive chromatin marks over mammalian gene terminators. Nature.

[48] Nissani N, Ulitsky I (2022). Unique features of transcription termination and initiation at closely spaced tandem human genes. Mol Syst Biol.

[49] Yang S, Zou Q, Liang Y, Zhang D, Peng L, Li W (2024). miR‐1246 promotes osteosarcoma cell migration via NamiRNA‐enhancer network dependent on Argonaute 2. MedComm.

[50] Bandiera S, Rüberg S, Girard M, Cagnard N, Hanein S, Chrétien D, Munnich A (2011). Nuclear outsourcing of RNA interference components to human mitochondria. PLoS One.

[51] Bian Z, Li LM, Tang R, Hou DX, Chen X, Zhang CY (2010). Identification of mouse liver mitochondria-associated miRNAs and their potential biological functions. Cell Res.

[52] Zhang X, Zuo X, Yang B, Li Z, Xue Y, Zhou Y (2014). MicroRNA directly enhances mitochondrial translation during muscle differentiation. Cell.

[53] Jagannathan R, Thapa D, Nichols CE, Shepherd DL, Stricker JC, Croston TL (2015). Translational regulation of the mitochondrial genome following redistribution of mitochondrial microRNA in the diabetic heart. Circ Cardiovasc Genet.

[54] Das S, Ferlito M, Kent OA, Fox-Talbot K, Wang R, Liu D (2012). Nuclear miRNA regulates the mitochondrial genome in the heart. Circ Res.

[55] Fan S, Tian T, Chen W, Lv X, Lei X, Zhang H (2019). Mitochondrial miRNA determines chemoresistance by reprogramming metabolism and regulating mitochondrial transcription. Cancer Res.

[56] Meseguer S, Rubio MP (2022). mt tRFs, new players in MELAS disease. Front Physiol.

[57] Shepherd DL, Hathaway QA, Pinti MV, Nichols CE, Durr AJ, Sreekumar S (2017). Exploring the mitochondrial microRNA import pathway through Polynucleotide Phosphorylase (PNPase). J Mol Cell Cardiol.

[58] Gao K, Cheng M, Zuo X, Lin J, Hoogewijs K, Murphy MP (2021). Active RNA interference in mitochondria. Cell Res.

[59] Wang WX, Visavadiya NP, Pandya JD, Nelson PT, Sullivan PG, Springer JE (2015). Mitochondria-associated microRNAs in rat hippocampus following traumatic brain injury. Exp Neurol.

[60] Vasudevan S, Tong Y, Steitz JA (2007). Switching from repression to activation: microRNAs can up-regulate translation. Science.

[61] Meseguer S, Navarro-González C, Panadero J, Villarroya M, Boutoual R, Sánchez-Alcázar JA (2019). The MELAS mutation m.3243A>G alters the expression of mitochondrial tRNA fragments. Biochim Biophys Acta Mol Cell Res.

[62] Zhan J, Jin K, Ding N, Zhou Y, Hu G, Yuan S (2023). Positive feedback loop of miR-320 and CD36 regulates the hyperglycemic memory-induced diabetic diastolic cardiac dysfunction. Mol Ther Nucleic Acids.

[63] Lopaschuk GD, Karwi QG, Tian R, Wende AR, Abel ED (2021). Cardiac energy metabolism in heart failure. Circ Res.

[64] Chen YR, Zweier JL (2014). Zweier. Cardiac mitochondria and reactive oxygen species generation. Circ Res.

[65] Bhullar SK, Dhalla NS (2023). Status of mitochondrial oxidative phospho-rylation during the development of heart failure. Antioxidants (Basel).

[66] Zhang X, Fan J, Li H, Chen C, Wang Y (2021). CD36 signaling in diabetic car-diomyopathy. Aging Dis.

[67] Rijzewijk LJ, van der Meer RW, Smit JW, Diamant M, Bax JJ, Hammer S (2008). Myocardial steatosis is an independent predictor of diastolic dysfunction in type 2 diabetes mellitus. J Am Coll Cardiol.

[68] Shu H, Peng Y, Hang W, Nie J, Zhou N, Wang DW (2022). The role of CD36 in cardiovascular disease. Cardiovasc Res.

[69] Chiu JJ, Chien S (2011). Effects of disturbed flow on vascular endothe-lium: pathophysiological basis and clinical perspectives. Physiol Rev.

[70] Luo M, Lu Z, Sun H, Yuan K, Zhang Q, Meng S (2010). Nuclear entry of active caspase-3 is facilitated by its p3-recognition-based specific cleavage activity. Cell Res.

[71] Duan H, Zhang Q, Liu J, Li R, Wang D, Peng W (2021). Suppression of apoptosis in vascular endothelial cell, the promising way for natural medicines to treat atherosclerosis. Pharmacol Res.

[72] Camargo LL, Rios FJ, Montezano AC, Touyz RM (2025). Reactive oxygen species in hypertension. Nat Rev Cardiol.

[73] Ding Y, Xia B, Yu J, Leng J, Huang J (2013). Mitochondrial DNA mutations and essential hypertension (Review). Int J Mol Med.

[74] Moulik M, Vatta M, Witt SH, Arola AM, Murphy RT, McKenna WJ (2009). ANKRD1, the gene encoding cardiac ankyrin repeat protein, is a novel dilated cardiomyopathy gene. J Am Coll Cardiol.

[75] Miller MK, Bang ML, Witt CC, Labeit D, Trombitas C, Watanabe K (2003). The muscle ankyrin repeat proteins: CARP, ankrd2/Arpp and DARP as a family of titin filament-based stress response molecules. J Mol Biol.

[76] Hang CT, Yang J, Han P, Cheng HL, Shang C, Ashley E (2010). Chromatin regulation by Brg1 underlies heart muscle development and disease. Nature.

[77] Sim CB, Phipson B, Ziemann M, Rafehi H, Mills RJ, Watt KI (2021). Sex-specific control of human heart maturation by the progesterone receptor. Circ.

[78] Wilcox NS, Amit U, Reibel JB, Berlin E, Howell K, Ky B (2024). Cardiovascular disease and cancer: shared risk factors and mechanisms. Nat Rev Cardiol.

[79] Herrmann J (2020). Adverse cardiac effects of cancer therapies: cardiotoxicity and arrhythmia. Nat Rev Cardiol.

[80] Palaskas NL, Ali HJ, Koutroumpakis E, Ganatra S, Deswal A (2024). Cardiovascular toxicity of immune therapies for cancer. BMJ.

